# Effects on heart function of neoadjuvant chemotherapy and chemoradiotherapy in patients with cancer in the esophagus or gastroesophageal junction – a prospective cohort pilot study within a randomized clinical trial

**DOI:** 10.1186/s13014-014-0310-7

**Published:** 2015-01-13

**Authors:** Mikael Lund, Gabriella Alexandersson von Döbeln, Magnus Nilsson, Reidar Winter, Lars Lundell, Jon A Tsai, Sigridur Kalman

**Affiliations:** Department of Anaesthesiology and Intensive Care, and Division of Anaesthesiology, CLINTEC, Karolinska Institutet, Karolinska University Hospital Huddinge, 141 86, Stockholm, Sweden; Department of Surgery and Division of Surgery, CLINTEC, Karolinska Institutet, Karolinska University Hospital Huddinge, 141 86, Stockholm, Sweden; Department of Clinical Oncology and Division of Oncology-Pathology, Karolinska Institutet, Karolinska University Hospital Solna, 171 76, Stockholm, Sweden; Department of Medicine, Section of Cardiology, and Division of Cardiology, Karolinska Institutet, Karolinska University hospital Huddinge, 141 86, Stockholm, Sweden; School of Technology and Health, Royal Institute of Technology, Stockholm, Sweden

**Keywords:** Esophageal neoplasms, Neoadjuvant therapy, Chemoradiotherapy, Echocardiography, Radiation effects, Cardiotoxicity

## Abstract

**Background:**

Neoadjuvant therapy for cancer of the esophagus or gastroesophageal (GE)-junction is well established. The pros and cons of chemoradiotherapy and chemotherapy are debated. Chemoradiotherapy might impair cardiac function eliciting postoperative morbidity. The aim of this pilot study was to describe acute changes in left ventricular function following chemoradiotherapy or chemotherapy.

**Methods:**

Patients with esophageal and (GE)-junction cancer enrolled at our center into a multicenter trial comparing neoadjuvant chemoradiotherapy and chemotherapy were eligible. Patients were randomized to receive cisplatin and 5-fluorouracil with or without the addition of 40 Gy radiotherapy prior to surgery. Left ventricular function was evaluated using echocardiography and plasma N-Terminal Pro-B-Type Natriuretic Peptide (NT-proBNP) before and after neoadjuvant treatment. The primary outcome measure was left ventricular global strain (GS). Clinical effects were assessed using repeated exercise tests. Linear mixed models were used to analyze the effects of treatment group, and the interaction between groups.

**Results:**

40 patients participated (chemoradiotherapy, n = 17; chemotherapy, n = 23). In the chemoradiotherapy group there was no change in left ventricular global strain but mitral annular plane systolic excursion (MAPSE) of the ventricular septum, early diastolic filling velocity (E-velocity), and the ratio of early to late ventricular filling velocities (E/A ratio) decreased significantly (p = 0.02, p = 0.01, and p = 0.03, respectively). No changes were observed in the chemotherapy group. There was a trend towards an interaction effect for MAPSE sept and E (p = 0.09 and p = 0.09). NT-proBNP increased following chemoradiotherapy (p = 0.05) but not after chemotherapy (p > 0.99), and there was a trend towards an interaction effect (p = 0.07). Working capacity decreased following neoadjuvant treatment (chemoradiotherapy p = 0.001, chemotherapy p = 0.03) and was more pronounced after chemoradiotherapy with a trend towards an interaction effect (p = 0.10).

**Conclusions:**

Neoadjuvant chemoradiotherapy but not chemotherapy before surgery for cancer of the esophagus or GE-junction seems to induce an acute negative effect on both systolic and diastolic left ventricular function. Future studies on neoadjuvant treatment for esophageal cancer are suggested to add measurements of cardiac function.

**Trial registration:**

Clinical Trials.gov NCT01362127.

**Electronic supplementary material:**

The online version of this article (doi:10.1186/s13014-014-0310-7) contains supplementary material, which is available to authorized users.

## Background

Neoadjuvant therapy has improved long-term survival after esophagectomy for cancer of the esophagus or gastroesophageal- (GE) junction even though long-term results are still poor [1]. Currently both chemotherapy and chemoradiotherapy are used. Two randomized trials have addressed the advantage of one regimen over the other [2,3]. Outcome results have, however, been criticized either due to small sample size, slow recruitment or lack of power to detect clinically relevant differences in outcomes. Neoadjuvant chemoradiotherapy has been suggested to increase postoperative mortality and possibly also morbidity [4,2,5], although these concerns were not confirmed according to other studies [6,7]. Currently it appears that chemotherapy alone does not increase postoperative morbidity or mortality while it is unclear whether chemoradiotherapy do.

Radiation therapy directed towards a tumor in the esophagus or GE-junction will inevitably irradiate the heart. Long-term side effects of modern thoracic radiotherapy include coronary artery disease and heart failure [8], but little is known about acute cardiac effects. Recent studies have demonstrated an acute decrease in left ventricular systolic and diastolic function following radiotherapy, but results are far from consistent [9-12]. Also N-Terminal Pro-B-Type Natriuretic Peptide (NT-proBNP), widely used to diagnose and prognose heart failure [13,14] has been observed to increase following radiotherapy [15]. The incidence of postoperative cardiac complications after esophagectomy is reported to be in the range of 15-30% for both chemo-and chemoradiotherapy [3,16,17].

The aim of this pilot study was to describe possible acute effects of neoadjuvant therapy on left ventricular function using echocardiography and NT-proBNP in patients with cancer of the esophagus or GE-junction. The primary outcome variable was global systolic left ventricular function measured as global strain (GS). The secondary outcome variables were ejection fraction (EF), regional systolic left ventricular function, diastolic function and NT-proBNP. In addition, general physical capacity was measured using repeated exercise test.

### Patients and methods

#### Patient inclusion

A multicenter randomized trial of neoadjuvant chemoradiotherapy versus chemotherapy for cancer in the esophagus or GE-junction was conducted between 2006 and 2013 in Sweden and Norway (NeoRes; EudraCTnr 2006-001785-16). The primary endpoint was the rate of complete histological response in the surgical specimen, which is a surrogate marker of improved long-term survival. The study was designed to include 180 patients based on a power calculation suggesting a sample size of 172 patients to achieve the primary endpoint assuming an increase in rate of complete histological response from 20% to 35% with a power of 80%. Inclusion criteria were age 75 years or less, histologically verified squamous cell carcinoma or adenocarcinoma of the esophagus or GE-junction, tumor stage T1-3 and N0-1 (according to AJCC TNM staging system 6th edition) [18], WHO performance status 0–1 and no major illness making neoadjuvant treatment unsuitable. Patients were stratified by histology before randomization. Randomization was done by a computer based program operational at the Regional Oncological Center in Stockholm.

The data presented here originate from a cohort of patients within the NeoRes trial scheduled for surgery at the Karolinska University Hospital. Patients underwent an extended protocol with studies of heart function which started in 2008. Added inclusion criteria were planned thoracoabdominal surgery. The NeoRes study protocol was approved by the regional ethics committee (EPN Stockholm 2006/738-32) and was registered at the registration site of the US National Institute of Health (ClinicalTrials.gov NCT01362127). For the extended protocol additional ethics approval was obtained (amendment 2008/1822-32). All patients were given written and oral information and were included after a signed informed consent had been obtained.

### Neoadjuvant treatment

Chemotherapy was given in three cycles of 21 days. Cisplatin 100 mg/m^2^ was given on day 1 and 5-fluorouracil 750 mg/m^2^/24 hrs was given on days 1–5. Cisplatin was switched to carboplatin or oxaliplatin in case of hearing impairment or renal dysfunction. Dose reduction was allowed for side effects. In the chemoradiotherapy group, concomitant radiotherapy was administered during cycles two and three with a total dose of 40 Gy; 2 Gy/day, 5 fractions per week. Radiotherapy was planned using a computer tomography-based three-dimensional treatment planning system (EclipseTM, Varian Medical systems, Palo Alto, USA). Treatment was administered using a multiple field technique with optimization of beam entry direction and beam weights in order to achieve a homogenous dose to planning target volume and minimize dose to organs at risk. Field shaping using a multi leaf collimator was used. For tumours located at or above the level of carina, the caudal border of the clinical target volume was 5 cm below diagnosed tumour, whereas the supraclavicular nodes defined the cranial border. For tumours located below the carina, the cranial border of the clinical target volume included 5 cm of radiographically uninvolved esophagus while the coeliac lymph nodes defined the caudal border, down to upper part of L1. In lateral, anterior and posterior directions clinical target volume encompassed gross tumour volume and the paraesophageal area with a margin of 1 cm but not including anatomical barriers as pleura, pericardium or bone. Dose–volume data of the heart including the pericardium with exclusion of the great vessels were extracted from individual dose distribution data in the treatment planning system. Planning target volume receiving >95% of planned radiation dose (PTV > 95%) all well as planning target volume receiving 95–105% of planned dose (PTV 95–105%) was measured. The percentage of the heart volume receiving 10 Gy or more (V10) and 30 Gy or more (V30) was measured. V30 was minimized as much as possible according to protocol. Surgery was scheduled 4–6 weeks after completion of neoadjuvant treatment.

### Echocardiography

Strain is a validated, relatively new, echocardiographic parameter for assessment of left ventricular function [19-21]. Strain gives an average of longitudinal shortening in the distance between individual speckles (natural acoustic markers) in the selected view of the ventricle. Global strain (GS) is thus the average strain of segments obtained from one projection. GS was chosen as the primary outcome parameter in this study as it is considered more sensitive for measuring left ventricular function and not as user dependent as EF [22] [23,24]. Other echocardiographic parameters were chosen in accordance with European Association of Echocardiography guidelines [25]. For regional function we measured atrioventricular movement using tissue Doppler, a robust parameter sensitive to early impairment of left ventricular longitudinal systolic function [26].

Echocardiography was performed before start of neoadjuvant therapy and repeated 4–6 weeks after completion of neoadjuvant therapy. A Vivid 7 ultrasound scanner (GE Vingmed, Horten, Norway) and a standard 2.5 MHz transducer were used. All but three exams were performed by the same laboratory technician according to research protocol. Post processing analysis was performed by a single analyser, ML, using Echo PAC (GE Vingmed, Horten, Norway) according to the manufacturer’s instructions. Both the echocardiography examiner and interpreter were blinded to study group allocation. All exams were interpreted after inclusion was closed.

**Figure 1:**
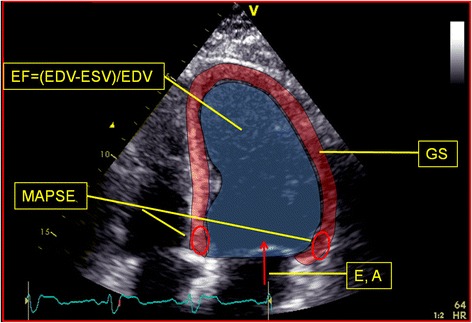
**Echocardiographic measurements.** Schematic illustration of echocardiographic measurements from apical four-chamber view. EF, ejection fraction measured according to Simpson Biplane; EDV, end diastolic volume; ESV end systolic volume; MAPSE, mitral annular plane systolic excursion; GS, global strain; E and A denotes blood velocities over the mitral valve during diastole.

A standard cardiac exam was performed on all subjects. One or more loops of three heartbeats were recorded online for each view and the best cardiac cycle selected for analysis during post processing. EF was calculated according to Simpson biplane. Mitral inflow was measured using pulsed wave Doppler. Peak velocity in the early rapid filling phase when the ventricle relaxes (E-Wave) and peak velocity of the late filling due to atrial contraction (A-Wave) were measured by pulsed Doppler across the mitral valve during diastole. Strain was measured using tissue tracing analysis in the apical four-chamber view centred on the left ventricle. For GS analysis a single cycle was used and all analyzed segments were approved by the program as well as by the analyzer. Tissue Doppler-based tissue tracing was used to measure atrio-ventricular plane movement. Mitral annular plane systolic excursion (MAPSE) was measured as the median value of three heart cycles using a region of interest (ROI) of 6×6 mm in the basal septum (sept) and basal anterolateral (lat) wall (Figure 1). Pictures were adjusted for optimal resolution and alignment of cardiac walls with the ultrasound beam.

### NT-proBNP

Venous blood was collected in EDTA tubes before neoadjuvant treatment and on admission for surgery 4–6 weeks after neoadjuvant therapy. Electrochemiluminescence immunoassay with Modular Analytics E170 (Roche Diagnostics, Mannheim, Germany) was used for the analyses, which were all performed by the Karolinska University Hospital accredited laboratory for clinical chemistry.

### Exercise test

Exercise tests of maximum work capacity measured in Watts were completed on a bicycle ergometer under the supervision of a dedicated staff member of an accredited physiology department. Maximum capacity was determined when patients were unable to maintain 60 revolutions per minute due to either, fatigue, shortness of breath or chest pain. The test could also be stopped by the staff due to the occurrence of arrhythmias, electrocardiogram changes or pathological blood pressure reactions. Testing was performed either at Karolinska University Hospital or at the patient’s referring hospital.

### Statistics

Data were analyzed as intention to treat using a linear mixed model analysis for repeated measures data. The model was used to analyze the effects of within-group change and the interaction between groups. This was defined as the basic model. In addition to the basic model, the influence of different covariates and factors was analyzed. The group effect was the difference between radiotherapy and chemotherapy, and the trend effect was the change between the first measure and the second measure. The within-group change was the difference between the first and second measure in respective groups. The interaction effect was the difference in change between groups. The covariance structure used in the models was unstructured. Before performing analyses, tests of normal distribution were performed and log transformation was performed for non-parametric data. Due to the small data set, several mixed models analyses were performed and in each analysis one of a set of covariates was added to the basic model to test for any impact on the results. The set of covariates was gender, age, BMI, ASA classification, hypertension, ischemic heart disease, smoking, chronic obstructive pulmonary disease, anemia, diabetes, and alcohol abuse. The Bonferroni correction was used to account for multiple comparisons. Patient characteristics are presented as median (range) and data regarding heart function as mean (95% confidence interval). Subject characteristics were analyzed using 2-tailed Mann Whitney U test and 2-tailed Fisher’s exact test as appropriate.

## Results

### Patient characteristics

**Figure 2:**
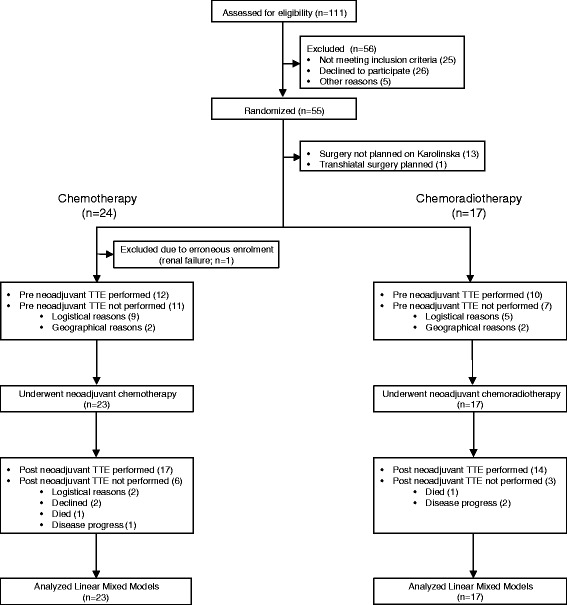
**Patient flow chart.** Flow chart of screened, enrolled and analyzed patients.

A total of 40 patients participated in the study (Figure 2). There were no statistically significant differences between the study groups regarding demographic and disease-specific characteristics as seen in Table 1. Six patients, (two in the chemoradiotherapy group) did not complete neoadjuvant treatment due to adverse events, persisting neutropenia in three cases, renal failure in one, peripheral neuropathy (foot drop) in one and circulatory instability in one. Five patients (two in the chemoradiotherapy group) had a reduced chemotherapy dose due to side effects. One patient had a reduced radiation dose due to thoracic pain. Target radiation dose was achieved in all other patients as shown by >95% and PTV 95–105%. Median PTV >95% was 99% (range 96–100%) and median PTV 95–105% was 97% (range 83–100%). Radiation dose to the heart was defined by V10 and V30. Median V10 to the heart was 74.9% (range 50.0–92.2%) and median V30 was 29.0% (range 0–80.1%).

**Table 1:** **Patient characteristics**

**Characteristics**	**Chemotherapy (n = 23)**	**Chemoradiotherapy (n = 17)**	**P**
**Age years median (range)**	62 (46–71)	66 (56–75)	0.09
**Male/Female**	19/4	15/2	1.00
**BMI median (range)**	23 (18–33)	26 (21–35)	0.06
**Weight change kg median (range)**	−10 (−24, +2)	−10 (−21, +6)	0.62
**Tumor type**			
**Adenocarcinoma, n (%)**	16 (70)	13 (76)	0.73
**Squamous-cell carcinoma, n (%)**	7 (30)	4 (24)	0.73
**Clinical T stage**			
**T1**	0	0	_
**T2**	9	4	_
**T3**	14	13	_
**Clinical N stage**			
**N0**	5	3	>0.99
**N1**	18	14	>0.99
**Cardiovascular disease, n (%)**	7 (30)	10 (59)	0.11
**Hypertension, n (%)**	7 (30)	9 (53)	0.20
**Ischemic heart disease, n (%)**	1 (4)	2 (12)	0.56
**Smoking ongoing or previous, n (%)**	10 (43)	4 (24)	0.32
**COPD, n (%)**	5 (22)	1 (6)	0.22
**Anemia, n (%)**	6 (26)	4 (23)	1.00
**Diabetes, n (%)**	3 (13)	3 (18)	1.00
**ASA class, n I/II/III/IV**	0/15/8/0	0/10/7/0	0.75

BMI, Body mass index; COPD, chronic obstructive pulmonary disease; ASA, American Society of Anesthesiologists.

### Echocardiography

Echocardiographic parameters were similar between groups before the start of neoadjuvant treatment. The neoadjuvant treatment induced no change in GS or EF in either of the groups (Table 2). MAPSE sept decreased significantly in the chemoradiotherapy group (mean change −1.4 mm, CI −2.4, −0.5, p = 0.02) and there was a trend towards an interaction effect (p = 0.09). E/A ratio in the chemoradiotherapy group also decreased significantly (mean change −0.22, CI −0.4, 0.1, p = 0.005) due to a significant decrease in E velocity (mean change −14.7 cm/s, CI −23.8, −5.8, p = 0.03) coupled with an unchanged A velocity (mean change +1.8 cm/s, CI −7.1, 10.7, p = 0.98). A trend towards an interaction effect was found for E velocity (p = 0.09) but not for E/A (p = 0.39). Adding the covariates gender, age, BMI, ASA classification, hypertension, ischemic heart disease, smoking, chronic obstructive pulmonary disease, anemia, diabetes and alcohol abuse to the model did not change the results significantly. See also Figure 2a-b in the additional file for scatterplots [Additional file 1]. No dose–response relationship was found between V30 and the echocardiographic changes (data not shown).

**Table 2:** **Results**

	**Chemotherapy**			**Chemoradiotherapy**			
**Variable**	**Pre neoadjuvant**	**Post neoadjuvant**	**P** ^**a**^	**Pre neoadjuvant**	**Post neoadjuvant**	**p** ^**a**^	**p** ^**b**^
EF (%)	59 (56–62)	57 (53–60)	>0.99	60 (57–64)	59 (55–63)	>0.99	0.80
GS (%)	−17.6 (−16, −19)	−15.7 (−14, −17)	0.26	−17.3 (−16, −19)	−16.1 (−14, −18)	>0.99	0.59
MAPSE sept (cm/s)	12.5 (11.5–13.5)	12.1 (11.2–13.1)	>0.99	12.6 (11.4–13.8)	11.1 (10.1–12.2)	0.02	0.09
MAPSE lat (cm/s)	11.5 (10.4–12.6)	11.2 (10.2–12.3)	>0.99	11.2 (10.0–12.4)	11.0 (9.8–12.1)	>0.99	0.96
E (cm/s)	72.0 (62.6–81.4)	68.1 (62.2–74.1)	>0.99	78.8 (68.4–89.3)	64.1 (57.2–70.9)	0.01	0.09
A (cm/s)	67.8 (58.2–77.5)	74.6 (63.9–85.3)	0.37	82.0 (71.1–92.7)	83.7 (71.6–95.9)	0.98	0.41
E/A	1.08 (0.93–1.25)	0.95 (0.81–1.10)	0.43	0.97 (0.82–1.14)	0.77 (0.65–0.92)	0.03	0.39
NT–ProBNP (ηg/l)	93 (58–149)	108 (70–167)	>0.99	65 (32–130)	154 (92–260)	0.05	0.07
Exercise test (W)	150 (135–165)	133 (115–151)	0.03	151 (133–151)	118 (96–140)	0.001	0.10

Data are presented as mean (95% confidence interval). ^a^Mixed models test of within-group changes; ^b^Mixed models test of interaction effect; EF, ejection fraction; GS, global strain; MAPSE, mitral annular plane systolic excursion; NT-proBNP, N-terminal pro-brain natriuretic peptide.

### NT-proBNP

After neoadjuvant treatment we observed a significant increase of NT-proBNP in the chemoradiotherapy group (mean 140%, CI 27–357%, p = 0.05), but no significant change after chemotherapy (mean 14%, CI 1–82%, p > 0.99) and a trend towards an interaction effect (p = 0.07) (Table 2). The addition of the covariates listed above did not change results significantly. See also Figure 2c in the additional file for scatterplot [Additional file 1]. No dose–response relationship was found between V30 and the NT-proBNP changes (data not shown).

### Exercise test

All tests were stopped by the patient due to fatigue or shortness of breath without indications of cardiac ischemia. Both neoadjuvant regimens were followed by a significant decrease in the patients working capacity (p = 0.03 and p = 0.001, respectively). This decrease was more pronounced in the chemoradiotherapy group (mean change −33 W, CI −48, −18 vs −17 W, CI −29, −5) but the interaction effect did not reach significance (p = 0.10) (Table 2). The addition of the covariates listed above did not change results significantly. See also Figure 2d in the additional file for scatterplot [Additional file 1].

## Discussion

Cardiac effects of neoadjuvant chemoradiotherapy in the treatment of cancer of the esophagus or GE-junction are important as they could have direct implications on anesthetic management and postoperative management as well as imply a need to individualize neoadjuvant treatment. This study provides results indicating that neoadjuvant chemoradiotherapy for cancer of the esophagus or GE-junction induces an acute impairment of heart function whereas neoadjuvant chemotherapy does not. Although we did not find any effect on our primary outcome variable global strain, a small but statistically significant decrease in septal function, was observed after chemoradiotherapy. The septum receives the highest radiation doses during chemoradiotherapy for esophageal cancer as shown by Hatakenaka et al. using magnetic resonance imaging [10]. In that study, regional wall motion was decreased for the mid anteroseptal, mid inferoseptal and mid inferior segments, which is in accordance with our findings. Moreover, in the study by Hatakenaka the largest decrease in wall movements was observed in the palliative patients who received the highest radiation doses indicating a dose-dependent response. Two retrospective studies have shown a decrease of EF after chemoradiotherapy using different combinations of chemotherapeutic agents and again using higher radiation doses (45–50 Gy) [11,27]. We were unable to show an effect on global systolic function, which may either be related to our small sample size or to the use of lower radiation doses.

Chemoradiotherapy also decreased the blood flow velocities over the mitral valve during the fast, passive filling phase of the left ventricle (E), coupled with an unchanged blood flow during atrial contraction (A) and accordingly a decreased E/A. These data suggest an impaired diastolic function as a consequence of impaired relaxation of the left ventricle. Hatakenaka and coworkers also reported an impairment of left ventricular relaxation after radiotherapy [10].

Neoadjuvant chemoradiotherapy also increased NT-proBNP. This biomarker has been studied as a predictor for the risk of perioperative cardiac complications with cutoff values between 201–791 ηg/ml being suggested [28,29]. Pre and perioperative levels of NT-proBNP are also strong predictors for atrial fibrillation even if cutoff levels are under debate [30].

We were unable to demonstrate a dose–response relationship between V30 and the echocardiographic changes or NT-proBNP levels. This may be due to the small number of patients enrolled but also dependent on the fact that V30 reflects the radiation dose targeting the whole heart rather than different segments. We refrained from doing a segmental analysis since this would require a larger dataset.

The clinical relevance of our findings is unclear. The level of systolic impairment detected was small and probably not clinically significant in a patient with an otherwise well-functioning left ventricle. Diastolic changes were larger with mean E/A levels after neoadjuvant therapy reaching grade I diastolic dysfunction which could have clinical implications. NT-proBNP levels increased following chemoradiotherapy and might indicate an increased risk for postoperative cardiac events and atrial fibrillation. This also shows that repeated NT-proBNP measurements could be a simple method to describe impairment from neoadjuvant treatment in future studies. Preoperative working capacity, as assessed during an exercise test, trajects into perioperative risks after esophagectomy [31,32]. We also observed that currently practiced neoadjuvant therapies, decreased the work capacity significantly in both groups. The effect was more pronounced in the chemoradiotherapy group but there was no significant interaction effect.

Cisplatin and 5-fluorouracil are commonly used drugs in chemotherapy regimens for esophageal cancer. These drugs are known to be associated with cardiotoxic side effects [33]. Therefore it was interesting to note that we were unable to detect echocardiographic or biochemical signs of decreased left ventricular function from chemotherapy alone.

The question whether neoadjuvant chemoradiotherapy is associated with an increased risk of postoperative morbidity compared with chemotherapy remains unresolved [5,7]. Only two previous randomized trials (n = 74 and n = 119) have directly compared neoadjuvant chemotherapy versus chemoradiotherapy [2,3]. However, functional studies of the heart were not a part of any of these prospective protocols.

Impaired left ventricular systolic and diastolic function following neoadjuvant chemoradiotherapy could well have a bearing on the incidence and grade of the innately high postoperative cardiovascular and pulmonary morbidity after esophagectomy. Taken together our data emphasize the relevance of dedicated studies aimed at further clarifying the details and consequences of the cardiotoxicity of current chemoradiotherapy regimens. One pathway that needs to be explored is cardiovascular function during the perioperative period.

One important limitation of our study is the small sample size, where the cases represented a cohort of patients consecutively enrolled from a larger, multicenter, randomized study. Two issues emerge as a consequence of this: the power of the observations and the possibility of selection bias. It should however, be emphasized that Karolinska University Hospital was the largest including center and the patients in our cohort represent 23% of the total study population. The randomization was not stratified to each center which may explain the small difference in the size of the treatment groups. There were no statistical differences in comorbidities between the study groups and the subsequent analysis of the effects of comorbidities in the linear mixed models did not display any significant impact on the results. Work capacity was also similar between the groups before neoadjuvant treatment suggesting comparable patients groups.

The second limitation, which we tried to confront, was the missing values within our cohort. The design of the present clinical study was very complex in a logistic perspective, in that we were faced with a short timeframe during which we had to plan and complete the echocardiographic examinations, i.e. from the randomization to the start of neoadjuvant treatment. During this limited time frame several other investigations and procedures (central lines for chemotherapy, PET-CT, endoscopic ultrasonography, respiratory and exercise test) had to be performed, usually at other hospitals. In addition, we chose to concentrate the echocardiography investigations at the tertiary referral center to increase the validity of the echocardiography. In order to partially compensate for the impact of missing data we adopted a linear mixed model statistics where the patients were analyzed as intention to treat. Linear mixed models give the opportunity to analyze all available data and not having to exclude cases where the dataset is incomplete. It also gives the option to correct for co-variables and is not restricted to a spherical or compound symmetry where we rely on the assumption that data are missing at random. By doing this we were able to mitigate some of the impact of missing data and further explore how patient comorbidity affected the results. No effect was found.

## Conclusions

Neoadjuvant chemoradiotherapy seems to induce a slight acute impairment of both systolic and diastolic left ventricle function, whereas chemotherapy does not. The systolic impairment was small and probably not clinically significant in a patient with an otherwise well-functioning left ventricle. The change in diastolic function was larger and might have clinical implications. These effects of chemoradiotherapy may enhance the risk of postoperative morbidity and should be taken into clinical consideration in patients with cardiac comorbidity. Corresponding and related effects on the heart need to be further explored in future studies of neoadjuvant chemoradiotherapy for cancer of the esophagus and GE-junction.

## Additional file

Additional file 1:
**Scatterplots of MAPSE lat, E, NT-proBNP and exercise capacity are shown.** All data are plotted with lines connecting paired measurements.

## References

[1] Sjoquist KM, Burmeister BH, Smithers BM, Zalcberg JR, Simes RJ, Barbour A (2011). Survival after neoadjuvant chemotherapy or chemoradiotherapy for resectable oesophageal carcinoma: an updated meta-analysis. Lancet Oncol..

[2] Stahl M, Walz MK, Stuschke M, Lehmann N, Meyer HJ, Riera-Knorrenschild J (2009). Phase III comparison of preoperative chemotherapy compared with chemoradiotherapy in patients with locally advanced adenocarcinoma of the esophagogastric junction. J Clin Oncol..

[3] Burmeister BH, Thomas JM, Burmeister EA, Walpole ET, Harvey JA, Thomson DB (2011). Is concurrent radiation therapy required in patients receiving preoperative chemotherapy for adenocarcinoma of the oesophagus? A randomised phase II trial. Eur J Cancer..

[4] Gignoux M, Bosset JF, Apoil B, Gillet M, Triboulet JP, Ollivier JM (1989). Adjuvant radiochemotherapy in operable cancers of the thoracic esophagus. Preliminary results of a multicenter study. A study of 119 cases. Ann Chir..

[5] Mariette C, Dahan L, Mornex F, Maillard E, Thomas PA, Meunier B (2014). Surgery alone versus chemoradiotherapy followed by surgery for stage I and II esophageal cancer: final analysis of randomized controlled phase III trial FFCD 9901. J Clin Oncol..

[6] Burmeister BH, Smithers BM, Gebski V, Fitzgerald L, Simes RJ, Devitt P (2005). Surgery alone versus chemoradiotherapy followed by surgery for resectable cancer of the oesophagus: a randomised controlled phase III trial. Lancet Oncol..

[7] van Hagen P, Hulshof MC, van Lanschot JJ, Steyerberg EW, van Berge Henegouwen MI, Wijnhoven BP (2012). Preoperative chemoradiotherapy for esophageal or junctional cancer. N Engl J Med..

[8] Jaworski C, Mariani JA, Wheeler G, Kaye DM (2013). Cardiac complications of thoracic irradiation. J Am Coll Cardiol..

[9] Erven K, Jurcut R, Weltens C, Giusca S, Ector J, Wildiers H (2011). Acute radiation effects on cardiac function detected by strain rate imaging in breast cancer patients. Int J Radiat Oncol Biol Phys..

[10] Hatakenaka M, Yonezawa M, Nonoshita T, Nakamura K, Yabuuchi H, Shioyama Y (2012). Acute cardiac impairment associated with concurrent chemoradiotherapy for esophageal cancer: magnetic resonance evaluation. Int J Radiat Oncol Biol Phys..

[11] Tripp P, Malhotra HK, Javle M, Shaukat A, Russo R, De Boer S (2005). Cardiac function after chemoradiation for esophageal cancer: comparison of heart dose-volume histogram parameters to multiple gated acquisition scan changes. Dis Esophagus..

[12] Nellessen U, Zingel M, Hecker H, Bahnsen J, Borschke D (2010). Effects of radiation therapy on myocardial cell integrity and pump function: which role for cardiac biomarkers?. Chemotherapy..

[13] Collinson PO, Gaze DC (2007). Biomarkers of cardiovascular damage and dysfunction–an overview. Heart Lung Circ..

[14] Wang TJ, Larson MG, Levy D, Benjamin EJ, Leip EP, Omland T (2004). Plasma natriuretic peptide levels and the risk of cardiovascular events and death. N Engl J Med..

[15] Jingu K, Nemoto K, Kaneta T, Oikawa M, Ogawa Y, Ariga H (2007). Temporal change in brain natriuretic Peptide after radiotherapy for thoracic esophageal cancer. Int J Radiat Oncol Biol Phys..

[16] Medical Research Council Oesophageal Cancer Working G (2002). Surgical resection with or without preoperative chemotherapy in oesophageal cancer: a randomised controlled trial. Lancet..

[17] Bosch DJ, Muijs CT, Mul VE, Beukema JC, Hospers GA, Burgerhof JG (2014). Impact of neoadjuvant chemoradiotherapy on postoperative course after curative-intent transthoracic esophagectomy in esophageal cancer patients. Ann Surg Oncol..

[18] Greene FL, Page DL, Fleming ID, Fritz A, Balch CM, Haller DG, Morrow M (2002). AJCC Cancer Staging Manual.

[19] Amundsen BH, Helle-Valle T, Edvardsen T, Torp H, Crosby J, Lyseggen E (2006). Noninvasive myocardial strain measurement by speckle tracking echocardiography: validation against sonomicrometry and tagged magnetic resonance imaging. J Am Coll Cardiol..

[20] Urheim S, Edvardsen T, Torp H, Angelsen B, Smiseth OA (2000). Myocardial strain by Doppler echocardiography. Validation of a new method to quantify regional myocardial function. Circulation..

[21] Urheim S, Rabben SI, Skulstad H, Lyseggen E, Ihlen H, Smiseth OA (2005). Regional myocardial work by strain Doppler echocardiography and LV pressure: a new method for quantifying myocardial function. Am J Physiol Heart Circ Physiol..

[22] Schiller NB, Shah PM, Crawford M, DeMaria A, Devereux R, Feigenbaum H (1989). Recommendations for quantitation of the left ventricle by two-dimensional echocardiography. American Society of Echocardiography Committee on Standards, Subcommittee on Quantitation of Two-Dimensional Echocardiograms. J Am Soc Echocardiogr..

[23] Hoffmann R, Lethen H, Marwick T, Arnese M, Fioretti P, Pingitore A (1996). Analysis of interinstitutional observer agreement in interpretation of dobutamine stress echocardiograms. J Am Coll Cardiol..

[24] Sitia S, Tomasoni L, Turiel M (2010). Speckle tracking echocardiography: A new approach to myocardial function. World J Cardiol..

[25] Evangelista A, Flachskampf F, Lancellotti P, Badano L, Aguilar R, Monaghan M (2008). European Association of Echocardiography recommendations for standardization of performance, digital storage and reporting of echocardiographic studies. Eur J Echocardiogr..

[26] Nikitin NP, Witte KK (2004). Application of tissue Doppler imaging in cardiology. Cardiology..

[27] Mukherjee S, Aston D, Minett M, Brewster AE, Crosby TD (2003). The significance of cardiac doses received during chemoradiation of oesophageal and gastro-oesophageal junctional cancers. Clin Oncol (R Coll Radiol)..

[28] Karthikeyan G, Moncur RA, Levine O, Heels-Ansdell D, Chan MT, Alonso-Coello P (2009). Is a pre-operative brain natriuretic peptide or N-terminal pro-B-type natriuretic peptide measurement an independent predictor of adverse cardiovascular outcomes within 30 days of noncardiac surgery? A systematic review and meta-analysis of observational studies. J Am Coll Cardiol..

[29] Ryding AD, Kumar S, Worthington AM, Burgess D (2009). Prognostic value of brain natriuretic peptide in noncardiac surgery: a meta-analysis. Anesthesiology..

[30] Cai GL, Chen J, Hu CB, Yan ML, Xu QH, Yan J (2014). Value of plasma brain natriuretic peptide levels for predicting postoperative atrial fibrillation: a systemic review and meta-analysis. World J Surg..

[31] Liedman B, Johnsson E, Merke C, Ruth M, Lundell L (2001). Preoperative adjuvant radiochemotherapy may increase the risk in patients undergoing thoracoabdominal esophageal resections. Dig Surg..

[32] Liedman BL, Bennegard K, Olbe LC, Lundell LR (1995). Predictors of postoperative morbidity and mortality after surgery for gastro-oesophageal carcinomas. Eur J Surg..

[33] Senkus E, Jassem J (2011). Cardiovascular effects of systemic cancer treatment. Cancer Treat Rev..

