# Supplementary material for: Effects on heart function of neoadjuvant chemotherapy and chemoradiotherapy in patients with cancer in the esophagus or gastroesophageal junction – a prospective cohort pilot study within a randomized clinical trial
Source: Radiat Oncol. 2015 Jan 13;10:16. doi: 10.1186/s13014-014-0310-7 (PMC4331444; doi:10.1186/s13014-014-0310-7)
Supplement: Additional file 1: — Scatterplots of MAPSE lat, E, NT-proBNP and exercise capacity are shown. All data are plotted with lines connecting paired measurements. [file 13014_2014_310_MOESM1_ESM.pdf]

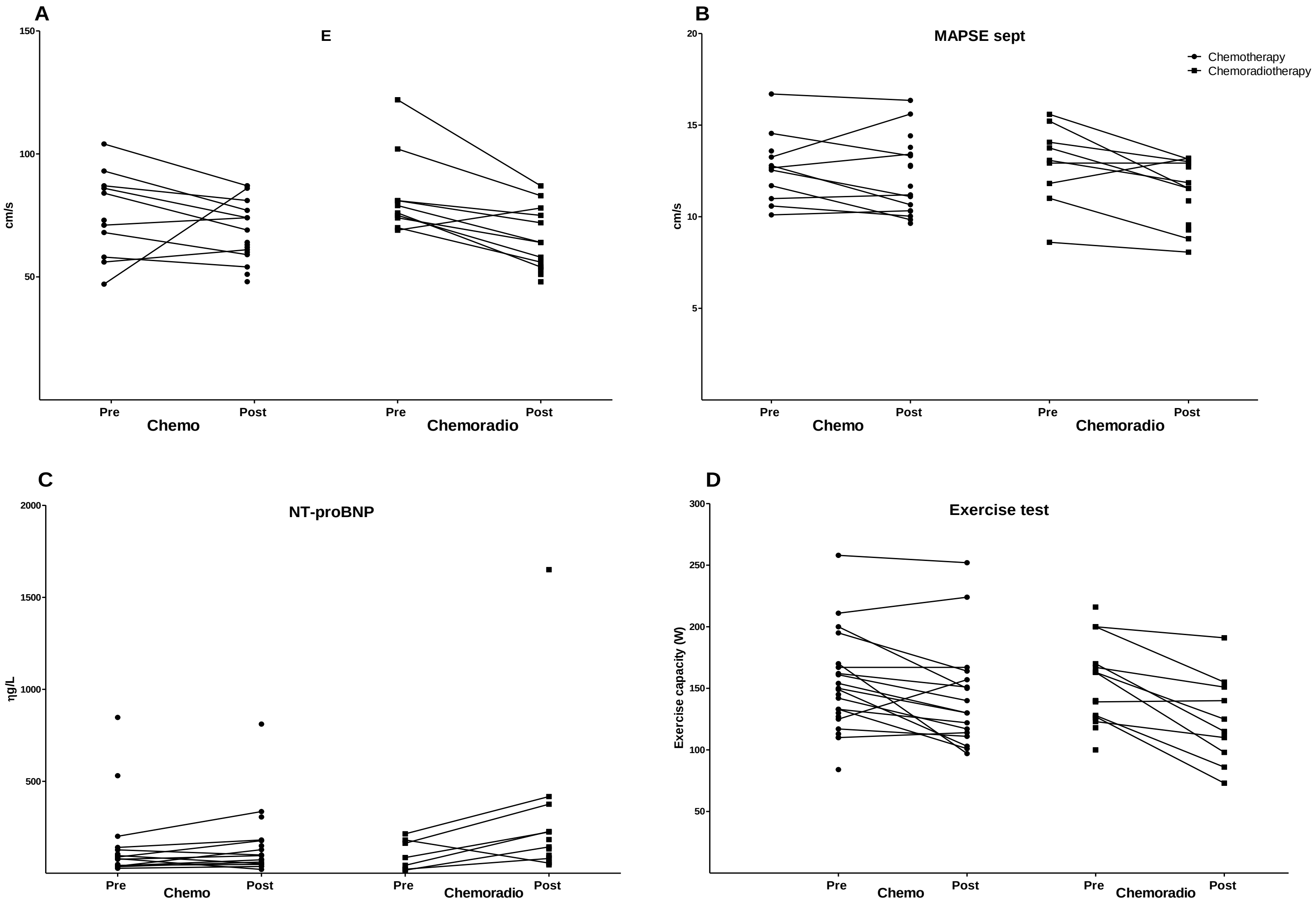

Fig 1. Scatterplotts of E (A), MAPSE sept (B), NT-proBNP (C) and exercise test (D) pre and post neoadjuvant treatment. Lines are connecting paired measurements
